# Ununited Fractures

**Published:** 1856-11

**Authors:** 


*Ununited Fractures.*

In the October number of this *Journal*, under the head of "Selections," we copied from some one of our exchanges the report of a discussion which took place in the New York Academy of Medicine on this subject. We did so chiefly for the purpose of adding some editorial comments, but in the multitude of business this was subsequently forgotten.

If the reader will turn to the October number, he will find Dr. Detmold, of New York, using the following language, viz.: "Five years ago Dr. D. had tried *boring* with a *common gimlet*, and was successful in all cases. A committee from the Academy had seen one of these operations. *This method was afterwards claimed by a Western Surgeon* (Dr. Brainard)."

This paragraph is not only erroneous, but doubly unjust. Dr. Brainard has never "claimed" any such operation as is described by Dr. Detmold. On the contrary, we find in the Introduction to his Prize Essay, published in the transactions of the American Medical Association, for 1854, the following explicit declaration, viz.:—"Others have proposed to make an incision, and attack the extremities (of the bone) *with a common gimlet;* an operation which has the double inconvenience—that it is liable to expose to suppuration, and that the *gimlet is incapable of perforating the compact structure of bone*, as any one can readily prove for himself by experiment."

Thus, instead of *claiming* the operation of Dr. Detmold as his own, Dr. Brainard distinctly states it as having been proposed by "*others*," and adds his objections to its practical application; and, hence, we were not a little surprised to see such a claim set up in the Academy of Medicine and reported for publication in the medical periodicals of the country without correction by any of the learned Fellows of that Society. To confound the attempts to perforate the ends of ununited bones with a "common gimlet," accompanied as it must be by free incisions through the soft parts—with the simple *sub-cutaneous perforation* with the neat instrument invented for that purpose, as proposed and practiced with the most gratifying success by Dr. Brainard, is just as absurd as to confound the free incisions of a common scalpel with the sub-cutaneous section of a tendon by a tenotomy-knife.

From this discussion in the New York Academy of Medicine, and from the allusion to the same subject in the recent volume of Dr. Smith on the Practice of Surgery, we think it would be useful to distribute a few copies of Dr. Brainard's Essay for the purpose of diffusing more accurate information.

